# The role of platelet derivatives in regenerative medicine: biological foundations and translational perspectives

**DOI:** 10.3389/fcell.2026.1770953

**Published:** 2026-03-31

**Authors:** Carlos Fernando Mourão, Gutemberg Gomes Alves

**Affiliations:** 1 Department of Clinical and Translational Research, Tufts University School of Dental Medicine, Boston, MA, United States; 2 Biology Institute, Cellular and Molecular Biology Department, Universidade Federal Fluminense, Niterói, Brazil

**Keywords:** dose delivered, human platelet lysate, platelet derivatives, platelet-rich fibrin, platelet-rich plasma, regenerative medicine, standardization

## Abstract

Platelet derivatives have become a central strategy in regenerative medicine due to their capacity to concentrate autologous signals that initiate and coordinate tissue repair. These materials function as transient regulators of the cellular microenvironment, shaping immune–stromal interactions, cell migration, differentiation programs, and extracellular matrix organization. However, terms such as platelet-rich plasma (PRP) and platelet-rich fibrin (PRF) encompass an ecosystem of preparations with considerable variability in composition, physical form, and biological performance. This heterogeneity, driven by differences in centrifugation parameters, anticoagulants, activation methods, tube types, and patient characteristics, explains inconsistent results across studies and hinders evidence consolidation. This mini-review discusses platelet derivatives along three axes: (i) their biological foundations and functional differences at the cellular and tissue microenvironment level among product families (PRP, PRF, formulations with modified plasma fractions, and human platelet lysate); (ii) how preparation variables impact reproducibility and interpretation of evidence; and (iii) current trends toward standardization, including classification systems, minimum reporting checklists, and dose/potency frameworks such as DEPA (Dose, Efficiency, Purity, Activation). Practical elements for minimum reporting are outlined to reduce product bias and enable cross-study comparisons. Ultimately, aligning study design with biological mechanisms and clinical indications through rational outcome selection will be essential to evaluate platelet derivatives as contemporary regenerative interventions with defined products, measurable quality, and clinically significant outcomes.

## 1 Introduction

Tissue repair and regeneration are fundamentally cell-driven processes, orchestrated by tightly regulated interactions between immune cells, stromal cells, extracellular matrix, and soluble mediators within a transient wound microenvironment. Platelet derivatives have emerged as regenerative strategies precisely because they concentrate and deliver, in autologous formulations, many of the early signals that initiate and modulate these cellular events (Calciolari et al., 2025; Miron et al., 2025). Platelet-rich plasma (PRP), platelet-rich fibrin (PRF) and their variations, as well as preparations combining modified plasma fractions with cellular concentrates (such as formulations based on denatured plasma combined with liquid PRF, as in Alb-PRF (Anselmo and Boselli, 2023)), represent distinct approaches in terms of physical form and release kinetics, yet converge on the same rationale: delivering mediators capable of modulating hemostasis, inflammation, angiogenesis, chemotaxis, and extracellular matrix remodeling, thereby fostering a microenvironment permissive to regeneration (Calciolari et al., 2025; Miron et al., 2025).

From a cellular perspective, the biological effects of platelet derivatives extend beyond the simplistic notion of increasing growth factor availability. Platelet activation shapes the wound microenvironment by influencing immune cell recruitment and polarization, stromal cell migration and proliferation, and the organization of a provisional extracellular matrix that guides early tissue patterning. They also interact with immune cells, including macrophages, particularly when leukocytes are part of the preparation (Anitua et al., 2024). Crucially, how these signals are presented to resident and recruited cells depends on the physical format of the platelet derivative. Liquid formulations tend to favor a more rapid diffusion and early signaling profile, whereas fibrin-rich matrices provide local, sustained mediator release (Calciolari et al., 2025) along with a scaffold for spatial organization of the healing tissue.

Despite widespread use and clinical enthusiasm, “platelet derivatives” do not describe a single intervention but rather an ecosystem of preparations with considerable variability in composition and biological performance, influenced by technical aspects of preparation and patient characteristics. This scenario explains the heterogeneity of results across studies and indications, supporting a paradigm shift: from the generic debate of “does it work or not?” to more informative and testable questions, such as “which preparation, at what dose and regimen, for which tissue, in what biological context, and in which patient profile?” (Calciolari et al., 2025; Anitua et al., 2024).

In this regard, recent research has prioritized strategies less focused on creating new nomenclatures and more aimed at optimizing specific properties for real regenerative needs (Calciolari et al., 2025; Miron et al., 2025; Miron et al., 2024). This mini-review discusses the role of platelet derivatives in regenerative medicine along three axes: (i) biological foundations and functional differences among the main product families (PRP, PRF, formulations associated with modified plasma fractions such as Alb-PRF, and HPL), (ii) how preparation and characterization variables influence reproducibility, comparability, and interpretation of evidence, and (iii) current trends toward more robust protocols, quality metrics, and study designs aligned with the mechanism and clinical indication. Finally, practical points are summarized to guide research and clinical translation, focusing on standardization, minimum reporting, and rational outcome selection, bringing platelet derivatives closer to the level of evidence expected for contemporary regenerative interventions.

## 2 Biological foundations and functional differences

Platelet derivatives share the same biological foundation: they reproduce, in concentrated form, the initial phase of tissue repair. Platelet activation triggers the release of mediators that regulate chemotaxis, cell proliferation and migration, angiogenesis, and extracellular matrix remodeling (Pinto et al., 2025). The central point for understanding the differences among families lies not only in the quantity of growth factors present but also in how the signal is delivered to the tissue, in terms of physical form, cellular content, and release kinetics (Calciolari et al., 2025).

PRP is, in essence, a platelet concentrate in liquid phase. It favors a more diffusible and generally earlier delivery of mediators, being particularly suitable when the primary goal is biochemical modulation of the microenvironment and initial repair stimulation in a well-vascularized site or in situations where no additional physical structure is required. As a liquid product, its function tends to depend more on the application regimen and preparation variables, including final platelet concentration and relative leukocyte content. In functional terms, the main characteristic of PRP is to act as a short-term “biological dose,” with less intrinsic contribution as a mechanical scaffold, unless it is converted into a gel, which can be achieved through activation (e.g., Calcium chloride (CaCl_2_), bovine thrombin, and contact with native collagen present in connective tissues) (Calciolari et al., 2025; Kawase et al., 2020; Gorodilova et al., 2024). Importantly, *in vitro* evidence indicates that increasing platelet-derived factor concentration directly enhances cellular migration and proliferation in a dose-dependent manner, underscoring the clinical relevance of achieving adequate platelet dosing in PRP preparations (Berger et al., 2019; Everts et al., 2024).

**FIGURE 1:**
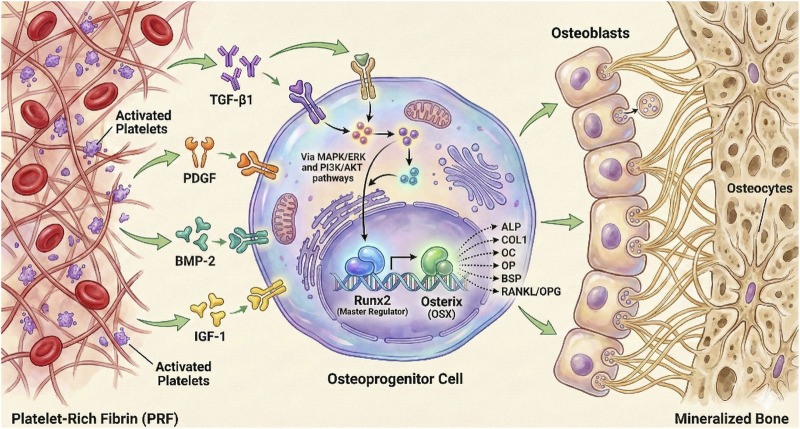
Schematic representation of PRF-mediated modulation of osteoprogenitor cell behavior and osteogenic differentiation. Activated platelets embedded within platelet-rich fibrin (PRF) release bioactive mediators, including PDGF, TGF-β1, BMP-2, and IGF-1, which act on osteoprogenitor cells within the local microenvironment. These signals engage intracellular pathways such as MAPK/ERK and PI3K/AKT, converging on osteogenic transcriptional regulators, particularly RUNX2 (CBFA1) and Osterix (OSX). Activation of these programs promotes the expression of matrix- and mineralization-associated genes (e.g., ALP, COL1, osteocalcin, BSP, and RANKL/OPG), supporting osteoblast differentiation and subsequent maturation into osteocytes within mineralized bone. This figure illustrates a conceptual model derived from scoping analyses of mineralizing cells, highlighting how PRF functions as a biologically active matrix that modulates defined cellular programs rather than acting as an unspecific growth factor mixture.

PRF, on the other hand, is defined by the presence of a three-dimensional fibrin matrix that entraps platelets and, in many variants, leukocytes. This architecture confers two relevant functional differences: greater local retention of bioactive content and more gradual release, in addition to a provisional scaffold role that promotes cell adhesion and initial organization of healing tissue. Thus, while PRP tends to operate as early-acting liquid signaling, PRF combines signaling and temporary structural support, which can be advantageous when local stability and biomaterial retention at the site are critical for the regenerative outcome (Miron et al., 2025; Malcangi et al., 2023). Scoping analyses focusing on mineralizing cells indicate that exposure to PRF membranes consistently activates signaling pathways associated with proliferation, osteogenic differentiation, and matrix-related gene expression (e.g., MAPK/ERK, TGF-β/SMAD and RUNX2-related transcriptional activity), supporting the concept that these platelet derivatives modulate defined cellular programs (de Lima Barbosa et al., 2023), as briefly described in Figure 1.

Formulations associated with modified plasma fractions, such as strategies based on heating platelet-poor plasma and recombining it with a cell-rich fraction (as in Alb-PRF, for example), emerge as a direct response to the temporal limitations of classic concentrates (Anitua et al., 2024). The objective here is to shift the product’s functional axis toward greater physical stability and longer residence time at the site, approaching a barrier and volume maintenance function without sacrificing the bioactivity conferred by the cellular fraction. The main difference, therefore, lies not in the platelet origin but in the biomaterial design: there is an explicit intention to modify the protein/plasma component to make it structurally more stable, delaying its degradation and extending local support compared to conventional PRF (Anselmo and Boselli, 2023; Kawase et al., 2020; Liu and Deng, 2022).

HPL (human platelet lysate) represents a distinct family because it is generally an acellular product based on content released through platelet lysis. The functional consequence is clear: HPL predominantly delivers soluble mediators, without an intrinsic fibrin matrix and without the structural variability typical of products formed by coagulation in the tube. For this reason, it fits better in regenerative medicine contexts that require greater predictability and quality control, such as cell expansion and tissue engineering applications, where the primary interest is a “cocktail” of factors under standardizable conditions rather than an autologous scaffold for immediate use (Anitua et al., 2024; Şeker et al., 2025; Burnouf et al., 2025). However, it is important to recognize that HPL, as a lysate-based product, delivers its entire bioactive content in a single bulk release, lacking the gradual, sustained release kinetics that characterize fibrin-embedded formulations *in vivo*. This distinction limits the temporal control over signaling and local retention at the recipient site that matrix-based derivatives inherently provide (Everts et al., 2024).

Increasing attention has also been given to the particulate fraction of platelet derivatives, particularly platelet-derived extracellular vesicles (EVs), as potential mediators of intercellular communication (Melki et al., 2017; Liu and Deng, 2022; Anselmo and Boselli, 2023). EVs represent a conceptual extension of platelet derivatives, as they may convey a more defined and concentrated set of bioactive signals capable of modulating recipient cell behavior without the structural and compositional complexity of fibrin-based matrices. This has raised interest in platelet-derived EVs as candidates for more targeted and potentially cell-free regenerative strategies. However, despite their biological plausibility, the translational relevance of these products remains limited by the lack of standardized protocols for isolation and characterization, the absence of robust dose–response and functional potency assays, and uncertainty regarding their stability and scalability as therapeutic products. As a result, EVs should currently be regarded as an emerging and exploratory component of platelet derivatives rather than a mature and reproducible regenerative intervention (Lou and Cai, 2025). Nonetheless, recent work on protein-rich, platelet-rich plasma (PR-PRP) matrices has highlighted the potential of autologous platelet-derived exosomes and microvesicles as integral components of the platelet secretome that may contribute to cell–cell communication and sustained reparative signaling within fibrin-based scaffolds (Everts et al., 2024).

## 3 Preparation and characterization variables: impact on reproducibility, comparability, and interpretation of evidence

The main barrier to consolidating evidence on platelet derivatives is not a lack of biological plausibility but rather the heterogeneity of the blood-derived product being tested. Two studies may indicate “PRP” or “PRF” and still evaluate biologically distinct interventions (Calciolari et al., 2025; Anitua et al., 2024) since, during preparation, small changes produce large differences. Centrifugation, for example, is the step that determines layer separation and depends on duration, speed, and relative centrifugal force (RCF); altering these parameters modifies the composition and the biological and mechanical properties of the concentrate (Anitua et al., 2024).

This variability is further amplified when anticoagulant, activation, handling time, and especially tube type come into play. For PRP, collection is typically performed in tubes containing anticoagulant (e.g., sodium citrate) (Kawase et al., 2020), which keeps the material in liquid phase during processing and allows subsequent activation, when desired, to induce gelation. For solid PRF (clot/membrane), tubes without anticoagulant are used, often made of glass or with a coagulation-activating surface (e.g., silica), which promotes rapid clot formation and influences fibrin architecture (Miron et al., 2025; Malcangi et al., 2023). For liquid PRF (i-PRF), tubes without additives or coagulation activators are typically employed, aiming to preserve injectability during the initial window before polymerization begins; in this scenario, variations of minutes between collection, centrifugation, and application can determine whether the material remains liquid or progresses to clot formation, impacting local retention, release kinetics, and scaffold function (Miron et al., 2020a; Miron et al., 2020b).

Comparability across studies is also compromised by the lack of minimum characterization of what was delivered to the tissue (Magalon et al., 2021; Al-Maawi et al., 2022). The inclusion or exclusion of leukocytes is a good example: studies show that this is often not even specified, which weakens any inference about immunomodulatory effects and hinders comparisons between products (Calciolari et al., 2025; Miron et al., 2025; Miron et al., 2024). Even when details are provided, processing conditions vary widely, and few studies compare, within the same experimental design, the same derivative “with” and “without” leukocytes, which would be the ideal scenario for attributing effects with greater confidence. Furthermore, different kits can generate products with distinct compositions, such as leukocyte-poor PRP versus leukocyte-rich PRP, which impacts cellular outcomes such as macrophage profiles (Anitua et al., 2024).

Evidence interpretation is affected because variability depends not only on the protocol but also on the donor and the biological context. Even when the preparation method is similar, patient characteristics such as age, comorbidities, and degree of systemic inflammation can alter the cellular and molecular profile of the derivative, influencing the balance of pro- and anti-inflammatory mediators and, consequently, the responses observed in experimental models and clinical applications (Anitua et al., 2024). Moreover, differences in outcome selection, assessment scales, and follow-up windows make cross-study comparisons less direct and increase uncertainty in interpreting effect size and clinical relevance of findings.

In practical terms, the message is simple: without rigorous description of preparation (centrifugation parameters, anticoagulant/activation, system used, and handling time) and without minimum product characterization (at least estimates of platelets, leukocytes, and residual erythrocytes, in addition to physical form and application method), the literature tends to group biologically different interventions under the same name. This reduces reproducibility, weakens comparability across studies, and hinders the translation of results into consistent clinical protocols (Calciolari et al., 2025; Anitua et al., 2024). These limitations reinforce that standardization of preparation and minimum product characterization are essential conditions for reproducibility and consistent interpretation of findings. This need parallels the growing recognition in the broader orthobiologic care field that every biological adjuvant, whether platelet derivatives or cell-based therapies, requires rigorous documentation of both the cellular and molecular components delivered to the patient (Moreno-Garcia and Rodriguez-Merchan, 2022; Winkler et al., 2025).

## 4 Current trends and future directions: standardization, quality, and rational study design

The clearest trends in the field today point toward transforming platelet derivatives into more comparable interventions, based on a common language and precisely described protocols. Recent reviews show that inconsistency in reporting preparation parameters contributes to conflicting conclusions and limits interpretation of individual studies (Calciolari et al., 2025; Miron et al., 2025; Miron et al., 2024), reinforcing the need for classification systems and detailed reporting to make results comparable. In this context, organization by “families” defined by cellular content (especially leukocytes) and fibrin architecture is gaining traction, distinguishing products such as PRP, solid PRF, and liquid PRF. Within these families, different subtypes (for example, Leukocyte-PRF and Advanced-PRF within solid PRF) reflect protocol variations without necessarily constituting biologically equivalent interventions (Calciolari et al., 2025; Masuki et al., 2016). This approach reduces the use of generic labels (broad terms without product specification) for biologically distinct interventions and facilitates replication and quantitative synthesis.

In parallel, there is a growing movement to adopt minimum quality and product characterization metrics as part of the study design itself. The absence of information about the preparation process and composition has been one of the main factors undermining reporting quality, and the considerable variability in composition and preparation generates a wide variety of biologically distinct products, making result comparison difficult.

Another important shift in the field is the move toward evaluating platelet derivatives closer within a potency and dose–response logic, rather than treating the product as a single block. It is still common for experimental designs not to adequately control concentration and exposure, but there is evidence that the effect may vary with derivative concentration, reinforcing the need to define the dose range and, when possible, include functional tests that serve as indirect potency indicators for the indication being studied (Calciolari et al., 2025; Miron et al., 2024; Masuki et al., 2016).

Finally, study design has become more aligned with the mechanism and clinical indication, with two practical implications. The first comes from choosing the type of derivative based on the biological objective: for example, when the goal is to maintain a scaffold/barrier for longer duration (as in facial harmonization or barrier functions), approaches to extend residence time emerge, such as the association to denatured albumin to prolong PRF resorption beyond weeks. The second is selecting comparators and outcomes consistent with the tissue and dominant mechanism, as illustrated in the literature on muscle injury comparing PRP and PPP, while recognizing that, despite distinct biological hypotheses, the lack of high-quality clinical trials still prevents definitive conclusions and firm guidelines. In the case of formulations such as Alb-PRF, the literature acknowledges promise but also highlights that clinical data remain scarce and that future studies need to clarify mechanisms and clinical efficacy, ideally with well-described protocols and appropriate comparators (Javid et al., 2023; Miron et al., 2024; Mourão et al., 2018; Fujioka-Kobayashi et al., 2021; Gheno et al., 2021).

Together, these trends converge toward a more “modern regenerative” standard: defining the product family and its critical parameters, minimally characterizing what is being delivered, incorporating dose/potency reasoning, and designing studies in which the choice of derivative and outcomes makes sense for the biology of the target tissue and the specific clinical requirement (Calciolari et al., 2025).

## 5 Discussion

To bring platelet derivatives closer to the level of evidence expected today, the first practical step is to stop treating “PRP” and “PRF” as sufficient labels and adopt harmonized classifications based on the preparation variables discussed above. In terms of clinical translation, this means that any study (and any center) must clearly state which product family is being used (e.g., PRP, solid PRF, liquid PRF, and formulations with modified plasma fractions such as Alb-PRF), how the product was obtained, and what “biological dose” was administered (dose delivered), that is, the combination of derivative concentration and volume applied (Calciolari et al., 2025; Şeker et al., 2025). This transparency enables replication by other groups and improves comparability across studies.

Minimum reporting should be brief but sufficient to reconstruct the product, avoiding common shortcomings identified in the literature, such as omissions of details about anticoagulant/activator, centrifugation conditions, and composition, which impact reporting quality and comparability. In practical terms, a minimum checklist should include, in the text or supplement: tube type and anticoagulant (if any), activation (if needed any), RCF/g-force and time of each centrifugation, rotor/system type, time between collection and centrifugation (critical for PRF), final volume obtained, and ideally a simple estimate of platelets, leukocytes, and residual erythrocytes in the product, preferably validated by automated hemocytometry at both baseline (whole blood) and in the final concentrate. This transparency reduces “product bias” and allows negative or positive results to be interpreted as effects of the derivative rather than effects of hidden protocol variation (Calciolari et al., 2025; Anitua et al., 2024; Kawase et al., 2020; Şeker et al., 2025). Table 1 summarizes these minimum reporting parameters.

**TABLE 1:** Minimum reporting checklist for platelet derivative studies.

Category	Parameter	Items to report
Collection	Anticoagulant	Type and concentration (e.g., 3.2% sodium citrate) or absence thereof
	Blood volume	Total mL collected
	Tube type	Material (glass/plastic), manufacturer, surface coating or additives
Processing	Centrifugation	RCF (×g), duration, number of spins, rotor type (fixed-angle/swing-out)
	Handling time	Intervals: collection→centrifugation→application
	Activation	Yes/No; if yes, agent and concentration (e.g., 10% CaCl_2_)
Final product	Product family	PRP/Solid PRF/Liquid PRF (i-PRF)/Alb-PRF/HPL, etc.
	Volume/dimensions	mL obtained or membrane dimensions (mm)
	Composition	Platelets, leukocytes, erythrocytes (×10^3^/µL or fold-increase vs. baseline); validated by automated hemocytometry at baseline and in the concentrate
Application	Physical form	Liquid/gel/membrane/minced
	Dose delivered	Volume applied × concentration
	Site and method	Injection, topical coverage, combined with biomaterial (specify)

The most mature trend in quality metrics is to quantify what was previously implicit. Instead of “rich PRP” or “strong PRP,” the proposal is to report dose and purity with a manufacturing logic: systems such as DEPA (Dose, Efficiency, Purity, Activation) (Calciolari et al., 2025; Magalon et al., 2016) make explicit the need to report the dose of platelets injected (concentration × volume), recovery efficiency, relative composition (platelets/leukocytes/erythrocytes), and whether activation occurred. For translational studies, this creates space for dose–response analyses and for incorporating simple “potency” tests aligned with the mechanism (e.g., a functional cell migration assay or a panel of released biological mediators), without turning the study into a large-scale laboratory project.

Finally, study design must be aligned with the mechanism (Magalon et al., 2021) and clinical indication (Pinto et al., 2025; Gheno et al., 2021), with outcomes chosen by logic rather than convenience. This begins with pre-specifying a primary outcome consistent with what the product can deliver during the time it remains active at the site, and with using realistic time windows for each family (earlier for PRP, more sustained for PRF and formulations with greater stability) (Javid et al., 2023; Miron et al., 2024; Mourão et al., 2018; Fujioka-Kobayashi et al., 2021; Gheno et al., 2021). It also involves selecting appropriate comparators and avoiding “variant inflation.” Although multiple modifications of the PRF protocol have been proposed, there is still no robust evidence that these adaptations generate clinically relevant differences, reinforcing the need for well-controlled and standardized trials rather than micro-variations that are difficult to compare. When this is done, platelet derivatives come to be evaluated as contemporary regenerative interventions: defined product, measurable minimum quality, and clinically significant and biologically plausible outcomes.

Thus, platelet derivatives remain a central platform in regenerative medicine, as they combine biological signaling and, in some families, matrix support, with practical feasibility across multiple clinical scenarios. To strengthen clinical translation, it is essential to define the product family, report minimum preparation and characterization parameters, and align outcomes and time windows with the kinetics of the derivative.
